# Post-COVID-19 Trends in Dental Emergencies: A Two-Year Retrospective Study from Romania

**DOI:** 10.3390/dj12120401

**Published:** 2024-12-09

**Authors:** Abel Emanuel Moca, Jessica Olivia Cherecheș, Lucian Roman Șipoș, Rahela Tabita Moca, Dan Slăvescu, Raluca Iurcov

**Affiliations:** Department of Dentistry, Faculty of Medicine and Pharmacy, University of Oradea, 10 Piața 1 Decembrie Street, 410073 Oradea, Romania; abelmoca@yahoo.com (A.E.M.); chereches_jessica@yahoo.com (J.O.C.); rahelamoca@gmail.com (R.T.M.); slavescudan@yahoo.com (D.S.); raluirimie@yahoo.com (R.I.)

**Keywords:** post-COVID-19, dental emergencies, Romania

## Abstract

**Background/Objectives**: Dental emergencies significantly impact public oral health, particularly in the post-COVID-19 context. This study aimed to analyze the patterns of dental emergencies presenting to the Emergency Dental Service in Bihor, Romania, during the years 2022 and 2023, focusing on demographic characteristics and the frequency of diagnoses. **Methods**: A retrospective analysis of medical records from the Emergency Dental Service at Oradea County Emergency Clinical Hospital was conducted. Inclusion criteria encompassed patients presenting with dental emergencies during the specified period. Data regarding demographics, diagnoses, and living environments were collected and statistically analyzed. **Results**: A total of 4769 patients were analyzed, with acute pulpitis (39.2%, *n* = 1869) and acute apical periodontitis (37.5%, *n* = 1788) identified as the most common diagnoses. The study population included 52.3% males and 47.7% females, with a larger proportion residing in urban areas (58.0%) compared with rural areas (42.0%). Significant age-related patterns were evident: pulpitis was more common among patients aged 10–39 years, trauma was associated with patients aged 0–9 years, and gingival infections were prevalent in the 70–79 age group. Diagnoses also varied by dentition type, with abscesses, caries, trauma, and rhizolysis occurring more frequently in deciduous teeth, while pulpitis and post-extraction alveolitis were predominant in permanent teeth. However, no statistically significant differences were found in diagnosis frequency between genders or between rural and urban patients, indicating equitable access to emergency services across environments. **Conclusions**: The findings underscore the need for targeted interventions in pediatric populations to address early childhood caries, which remains a significant burden. Enhanced public health strategies and preventive education are essential to mitigate the prevalence of dental emergencies, particularly in the aftermath of the COVID-19 pandemic.

## 1. Introduction

Dental emergencies, commonly involving pain, infection, and trauma, frequently present in hospital emergency departments [1]. While typically not life-threatening, they can cause significant pain and impact dental aesthetics, quality of life, and overall well-being [2,3]. Given their high prevalence [4] and negative consequences, dental emergencies are a critical public oral health concern [3].

Most dental emergencies result from untreated dental caries, with 3.09 billion new cases of untreated caries in permanent teeth reported globally in 2019 [5]. Irreversible pulpitis, a common consequence, causes intense pain that typically doesn’t respond to analgesics [6,7], requiring immediate intervention and long-term endodontic treatment [8]. If untreated, pulpitis can progress to acute apical periodontitis, a complication of pulp necrosis or inflammation [9]. Dental abscesses, while often resolving through spontaneous drainage, can spread, posing life-threatening risks and requiring prompt intervention [10].

Dento-periodontal traumas are diverse and exhibit a relatively high prevalence among the adult population, with approximately 30% of adults experiencing some form of dental trauma. Consequently, they are recognized as a significant community oral health issue [11]. These traumas range from dislocations and fractures that do not expose the pulp chamber to fractures with pulp chamber involvement, root fractures, and, in the most severe cases, tooth avulsion [12]. Given their frequent occurrence in the anterior teeth, the resulting aesthetic deficits are substantial, compounded by pain and functional impairment [12].

During the COVID-19 pandemic in Romania, when dental offices were limited to emergency treatments, the Romanian College of Dentists recognized pathologies such as post-extraction hemorrhages, pulpitis, apical periodontitis, and abscesses as dental emergencies [13]. Despite restrictions, dental emergency visits remained high [4]. Implementing COVID-19 testing offered a sustainable strategy for safely resuming dental services, as it helped identify asymptomatic carriers and minimized the risk of cross-infection [14]. Although the World Health Organization declared the end of the global COVID-19 public health emergency on 5 May 2023 [15], Romania relaxed restrictions earlier, on 9 March 2022, marking a return to normal healthcare operations [16]. This study, covering 2022 and 2023, reflects trends as dental services transitioned back to routine care.

Post pandemic, it is essential to assess the impact of relaxed measures [17] and update the overview of emergency dental services, especially given the high prevalence of dental caries [18] and reliance on emergency care [19] in the population. Given these aspects, it was hypothesized that the frequency and types of dental emergencies in Bihor, Romania, increased in the post-COVID-19 period due to delays in treatment caused by pandemic-related restrictions, with certain demographic groups, particularly those with untreated dental caries, being at higher risk for more severe dental conditions.

This study aims to investigate the patterns of dental emergency visits in Bihor, Romania, during 2022 and 2023, specifically focusing on the types of emergencies, demographic factors, and the frequency of presentations in the post-COVID-19 era. The research seeks to answer the following questions: (1) How have the types and frequencies of dental emergencies changed after the easing of COVID-19 restrictions? (2) Are there specific demographic groups at higher risk of presenting with untreated dental conditions in this period? By addressing these questions, the study will provide insights that can inform public health strategies for reducing the burden of dental emergencies and targeting preventive interventions.

## 2. Materials and Methods

### 2.1. Ethical Considerations

This study was conducted in accordance with the ethical principles outlined in the Declaration of Helsinki (2008), including subsequent amendments. Approval was obtained from the Ethics Committee of the Oradea County Emergency Clinical Hospital (IRB No. 22143/From 6 July 2022). Upon presentation at the dental emergency service, adult patients (aged 18 and older) provided written informed consent, permitting the anonymous use of their medical data for future scientific research. For patients under the age of 18, informed consent for the anonymized use of medical data was obtained from their parents or legal guardians.

### 2.2. Participants and Data Collection

This retrospective study was conducted by analyzing the medical records of patients treated for urgent cases at the Emergency Dental Service of the Oradea County Emergency Clinical Hospital. This service, which is free of charge, operates within the dental clinic of the Faculty of Medicine and Pharmacy at the University of Oradea. Emergency dental cases are treated around the clock, without the need for prior appointments, with a dentist and nurse available at all times. For this study, medical records from patients treated in 2022 and 2023, following the conclusion of COVID-19 restrictions in Romania, were analyzed.

Since this study was retrospective in nature, no randomization procedure was performed; instead, all patients meeting the criteria were included to provide a comprehensive overview of dental emergencies during this time. Initially, all medical records from patients who visited the emergency dental service in 2022 and 2023 were included. However, after reviewing the records, certain exclusions were made. These encompassed records missing relevant information (e.g., gender, age, affected tooth, diagnosis, treatment), cases where patients refused treatment, those marked as uncooperative and unable to be treated chair-side, and patients requiring maxillofacial surgery who were referred to the Oral and Maxillofacial Surgery Department of the Oradea County Emergency Clinical Hospital. Additionally, patients whose diagnoses did not constitute medical emergencies (e.g., oral thrush, dental eruptions, unfinished fillings) were also excluded.

This study considered the following variables: gender (male, female), age (grouped as 2–9 years, 10–19 years, 20–29 years, 30–39 years, 40–49 years, 50–59 years, 60–69 years, 70–79 years, 80–89 years), living environment (urban, rural), dentition (temporary, permanent), and the diagnosis received during the emergency dental examination. The recorded emergencies were as follows: acute pulpitis (characterized by severe, persistent tooth pain due to inflammation of the dental pulp), acute apical periodontitis (often a progression from untreated pulpitis, this condition involves inflammation and infection of the tissues surrounding the apex of a dental root), dental abscess (localized collections of pus resulting from bacterial infections in the soft tissues), post-extraction alveolitis (a painful complication following tooth extraction, typically caused by the dislodgement of the blood clot protecting the socket), post-extraction hemorrhage (excessive bleeding following tooth extraction), pericoronitis (inflammation of the soft tissues around a partially erupted tooth, most commonly associated with third molars), dento-alveolar trauma (injuries to the teeth and surrounding structures, including dislocations, fractures, and avulsions), periodontal emergencies (conditions such as acute gingival or periodontal abscesses causing pain and swelling, often accompanied by mobility of affected teeth), radicular resorption in deciduous teeth (a natural process in children as permanent teeth begin to erupt, but occasionally presenting as a dental concern if associated with pain or infection).

To minimize bias, the medical records were independently reviewed by both the author responsible for data collection and the author responsible for compiling the statistics.

### 2.3. Statistical Analysis

Statistical analyses were conducted using IBM SPSS Statistics version 25 and Microsoft Office Excel/Word 2013. Qualitative variables, such as gender, living environment, and diagnosis, were summarized as absolute values and percentages to provide a clear, descriptive overview of the sample characteristics. To assess associations between categorical variables, Fisher’s exact test was chosen due to its robustness with small sample sizes and its suitability for examining relationships between categorical data. This test ensures that the analysis remains valid even when the data includes cells with low expected frequencies.

To further investigate significant associations, Z-tests with Bonferroni correction were employed. This approach was selected to control for Type I errors that may arise from multiple comparisons in the contingency tables, thereby ensuring the reliability and accuracy of the findings. These methods were deemed appropriate given the retrospective nature of the study and the need to analyze categorical data to identify trends in dental emergencies by demographic characteristics and living environments. The combination of these statistical tests directly aligns with the study’s goals of identifying patterns and drawing reliable conclusions about the prevalence and distribution of dental emergencies in the population.

## 3. Results

### 3.1. Sample Characteristics

**Figure 1:**
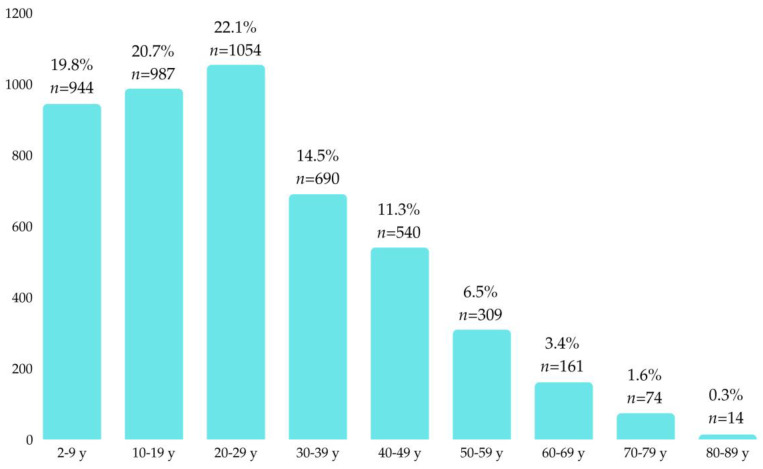
Distribution of patients by age.

After applying inclusion and exclusion criteria, 4769 patients were included in the study, with 52.3% (*n* = 2492) male and 47.7% (*n* = 2277) female. Most patients (58.0%, *n* = 2766) resided in urban areas, while 42.0% (*n* = 2001) were from rural settings. The largest age group was 20–29 years (22.1%, *n* = 1054), followed by 10–19 years (20.7%, *n* = 987), while the smallest group was 80–89 years (0.3%, *n* = 14), as shown in Figure 1.

During the years 2022 and 2023, a total of 4773 teeth were treated in the dental emergency service. Of these, 904 (18.9%) were deciduous teeth, and 3869 (81.1%) were permanent teeth. The detailed distribution of affected teeth by tooth group is presented in Table 1.

**Figure 2:**
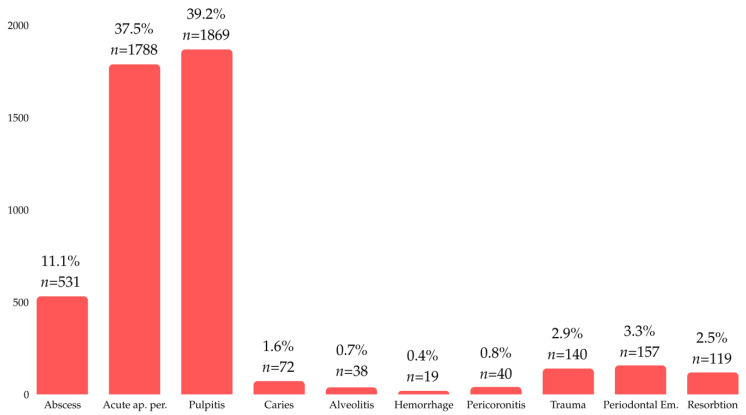
Distribution of patients by diagnoses.

The most frequent diagnoses were acute pulpitis (*n* = 1869, 39.2%) and acute apical periodontitis (*n* = 1788, 37.5%) (Figure 2).

### 3.2. The Influence of Gender, Age, Living Environment, and Dentition on Diagnosis

Table 2 illustrates the distribution of patients by diagnosis and gender. Fisher’s exact test revealed no significant differences between male and female patients (*p* = 0.444), indicating similar diagnosis frequencies across genders.

Significant age-related differences were observed (*p* < 0.001) based on Z-tests with Bonferroni correction. Pulpitis was more frequent in the 10–39-year age groups, while trauma and radicular resorption of deciduous teeth were prevalent in patients aged 0–9 years. Gingival infections were notably higher in the 70–79-year group (Table 3).

As with gender, no significant differences in diagnosis frequency were observed between rural and urban patients (*p* = 0.150, Table 4), indicating similar patterns across living environments.

Table 5 shows the distribution of diagnoses by dentition type (temporary vs. permanent). Significant differences were found (*p* < 0.001). Abscesses (13.4% vs. 10.6%), caries (2.7% vs. 1.2%), trauma (4% vs. 2.7%), and rhizolysis (8.3% vs. 1.1%) were more frequent in deciduous teeth. In contrast, pulpitis (41.1% vs. 30.8%) and post-extraction alveolitis (1% vs. 0.1%) were more common in permanent teeth.

## 4. Discussion

The findings of this study provide a comprehensive analysis of dental emergencies presented to the Emergency Dental Service in Bihor, Romania, during 2022 and 2023. As anticipated, the most frequent diagnoses were acute pulpitis and acute apical periodontitis, conditions closely associated with untreated dental caries [5,20], aligning with global patterns in dental emergencies [2,21]. A study conducted by Huang et al. (2022) on a population group in southern Taiwan reported similar results regarding the most frequently encountered diagnoses, with pulp-related pathology being the most common condition identified [22]. The data regarding the prevalence of these two pathologies also support the findings obtained in this study. Concerning acute apical periodontitis, a systematic review and meta-analysis conducted by Tibúrcio-Machado et al. (2021) reported a global prevalence of 52% at the individual level [23]. Although it is difficult to conduct a systematic review for pulpitis, particularly because the diagnosis can be unreliable [24], epidemiological studies on different population groups have identified values similar to those obtained in this study [25,26].

Both conditions were similarly prevalent in male and female patients, with no statistically significant differences by gender, corroborating previous studies that highlight the equal susceptibility of both sexes to dental emergencies stemming from untreated caries [27]. The data indicated a notable association between age and certain types of dental emergencies. Acute pulpitis, for instance, was significantly more frequent in younger age groups, particularly those aged 10–39 years, while trauma was more commonly observed in children aged 0–9 years. This pattern is consistent with findings from other studies, which suggest that younger populations are at greater risk of dental caries due to inadequate oral hygiene practices and less frequent dental visits [28,29]. Supporting these results, Soh et al. (2021) identified a high prevalence of pulpitis even in primary teeth [30]. Furthermore, the high incidence of trauma in young children can be attributed to increased physical activity and accidents, which are well-established causes of dental trauma in pediatric populations [31].

Additionally, the study highlighted the disparity between urban and rural populations in terms of the frequency of dental emergencies, although no statistically significant differences were found. This observation reflects broader socio-economic challenges in rural communities, where access to routine dental care may be limited [32]. Previous research has documented similar trends, suggesting that individuals in rural areas are more likely to seek emergency dental services for conditions that could have been prevented through regular check-ups [4,33]. The lack of significant variation in diagnosis frequency between rural and urban groups in this study could be attributed to the universal accessibility of the emergency dental service at Oradea County Emergency Clinical Hospital, where services are provided free of charge and without the need for prior appointments.

Another key finding of this study is the significant prevalence of abscesses in deciduous teeth among children. These conditions were disproportionately more frequent in deciduous dentition compared with permanent teeth, reinforcing the need for targeted pediatric dental interventions. Early childhood caries is a significant burden in most European Union countries and is often left untreated, with negative effects on the quality of life of patients, as well as on the development of the dento-maxillary apparatus and overall growth [34]. Globally, the incidence of early childhood caries is 1.76 billion, and it is not limited to children from disadvantaged socio-economic backgrounds [35]. Given the rapid progression of this pathology [35], early diagnosis and treatment of dental caries in children could prevent the progression to more severe conditions such as abscesses, which are associated with higher morbidity and the potential for life-threatening complications if left untreated [36].

The findings of this study on post-COVID-19 trends in dental emergencies align with those of Bennardo et al. (2020), who analyzed changes in outpatient oral surgery during the COVID-19 pandemic in an Italian center. Their study highlighted a significant reduction in routine dental care, with a focus on urgent cases such as abscesses, bacterial infections, and trauma, which mirrors the prevalence of similar emergencies observed in our study. While Bennardo et al. implemented strict triage protocols and remote consultations to manage care during the pandemic, our findings emphasize the lingering impact of deferred routine care, as reflected in the high incidence of acute pulpitis and apical periodontitis in the post-pandemic period [37]. These parallels underscore the need for robust public health strategies to address the backlog of untreated dental conditions and prevent their progression to emergencies.

This study captures trends in dental emergencies during the 2022–2023 period; however, it remains unclear whether these patterns are unique to the post-pandemic period or reflective of pre-existing trends, as highlighted by previous Romanian studies. The retrospective design also did not evaluate whether certain conditions unique to the COVID-19 period were recorded in patient records. Additionally, the assessment of radicular resorption relied on patient records rather than direct analysis of X-ray images, which could limit accuracy. Finally, the single-center nature of the study restricts generalizability, and reliance on retrospective data may introduce reporting bias.

## 5. Conclusions

This study highlights the high prevalence of acute pulpitis and apical periodontitis in post-COVID-19 dental emergencies, emphasizing the need for targeted population care and improved access to preventive services. The findings underscore the importance of addressing untreated dental conditions through public health initiatives and integrating robust infection control measures, such as COVID-19 testing, to ensure sustainable and safe dental care delivery.

## Figures and Tables

**Table 1:** Distribution of affected teeth.

Group of Teeth	No.	%
**Permanent Dentition**		
Incisors	384	9.9%
Canines	222	5.7%
Premolars	941	24.3%
Molars	2322	60.1%
**Total**	3869	100.0%
**Deciduous Dentition**		
Incisors	152	16.8%
Canines	22	2.4%
Molars	730	80.8%
**Total**	904	100.0%

**Table 2:** Distribution of Patients by Diagnosis and Gender.

Diagnosis	Female		Male		*p* *
	No.	%	No.	%	
Abscess	260	11.4%	271	10.9%	0.444
Acute apical periodontitis	853	37.5%	933	37.4%	
Pulpitis	873	38.3%	994	39.9%	
Caries	37	1.6%	35	1.4%	
Post-extraction alveolitis	19	0.8%	19	0.8%	
Post-extraction hemorrhage	6	0.3%	13	0.5%	
Pericoronitis	19	0.8%	21	0.8%	
Trauma	70	3.1%	70	2.8%	
Periodontal emergencies	88	3.9%	69	2.8%	
Radicular resorbtion in deciduous teeth	52	2.3%	67	2.7%	

* Fisher’s Exact Test.

**Table 3:** Distribution of Patients by Diagnosis and Age.

Diagnosis	2–9	10–19	20–29	30–39	40–49	50–59	60–69	70–79	80–89	*p* *
	No. (%)	No. (%)	No. (%)	No. (%)	No. (%)	No. (%)	No. (%)	No. (%)	No. (%)	
Abscess	122 (12.9%)	106 (10.7%)	102 (9.7%)	81 (11.7%)	61 (11.3%)	31 (10.0%)	18 (11.2%)	8 (10.8%)	2 (14.3%)	<0.001
Acute apical periodontitis	345 (36.5%)	346 (35.1%)	386 (36.6%)	270 (39.1%)	217 (40.2%)	119 (38.5%)	70 43.5%)	28 (37.8%)	7 (50.0%)	
Pulpitis	303 (32.1%)	420 (32.6%)	469 (44.5%)	285 (41.3%)	199 (36.9%)	118 (38.2%)	53 (32.9%)	21 (28.4%)	1 (7.1%)	
Caries	25 (2.6%)	13 (1.3%)	14 (1.3%)	8 (1.2%)	5 (0.9%)	2 (0.6%)	2 (1.2%)	3 (4.1%)	0 (0.0%)	
Post-extraction alveolitis	3 (0.3%)	8 (0.8%)	11 (1.0%)	7 (1.0%)	4 (0.7%)	3 (1.0%)	1 (0.6%)	0 (0.0%)	1 (7.1%)	
Post-extraction hemorrhage	2 (0.2%)	1 (0.1%)	5 (0.5%)	2 (0.3%)	5 (0.9%)	2 (0.6%)	1 (0.6%)	1 (1.4%)	0 (0.0%)	
Pericoronitis	5 (0.5%)	13 (1.3%)	13 (1.2%)	5 (0.7%)	2 (0.4%)	1 (0.3%)	0 (0.0%)	1 (1.4%)	0 (0.0%)	
Trauma	42 (4.4%)	26 (2.6%)	30 (2.8%)	11 (1.6%)	11 (2.0%)	10 (3.2%)	6 (3.7%)	3 (4.1%)	1 (7.1%)	
Periodontal emergencies	33 (3.5%)	30 (3.0%)	14 (1.3%)	13 (1.9%)	27 (5.0%)	21 (6.8%)	8 (5.0%)	9 (12.2%)	2 (14.3%)	
Radicular resorbtion	64 (6.8%)	24 (2.4%)	10 (0.9%)	8 (1.2%)	9 (1.7%)	2 (0.6%)	2 (1.2%)	0 (0.0%)	0 (0.0%)	

* Fisher’s exact test.

**Table 4:** Distribution of Patients by Diagnosis and Living Environment.

Diagnosis	Rural		Urban		*p* *
	No.	%	No.	%	
Abscess	229	11.4%	302	10.9%	0.150
Acute apical periodontitis	749	37.4%	1036	37.5%	
Pulpitis	782	39.1%	1085	39.2%	
Caries	30	1.5%	42	1.5%	
Post-extraction alveolitis	23	1.1%	15	0.5%	
Post-extraction hemorrhage	6	0.3%	13	0.5%	
Pericoronitis	16	0.8%	24	0.9%	
Trauma	68	3.4%	72	2.6%	
Periodontal emergencies	55	2.7%	101	3.7%	
Radicular resorbtion in deciduous teeth	43	2.1%	76	2.7%	

* Fisher’s exact test.

**Table 5:** Distribution of Patients by Diagnosis and Dentition.

Diagnosis	Deciduous		Permanent		*p* *
	No.	%	No.	%	
Abscess	121	13.4%	410	10.6%	<0.001
Acute apical periodontitis	337	37.3%	1451	37.5%	
Pulpitis	278	30.8%	1591	41.1%	
Caries	24	2.7%	48	1.2%	
Post-extraction alveolitis	1	0.1%	37	1.0%	
Post-extraction hemorrhage	1	0.1%	18	0.5%	
Pericoronitis	5	0.6%	35	0.9%	
Trauma	36	4.0%	104	2.7%	
Periodontal emergencies	26	2.9%	131	3.4%	
Radicular resorbtion in deciduous teeth	75	8.3%	44	1.1%	

* Fisher’s exact test.

## Data Availability

The data presented in this study are available on request from the corresponding authors. The data are not publicly available due to privacy reasons.

## References

[1] Hammel J.M., Fischel J. (2019). Dental emergencies. Emerg. Med. Clin. N. Am..

[2] Loureiro R.M., Naves E.A., Zanello R.F., Sumi D.V., Gomes R.L.E., Daniel M.M. (2019). Dental emergencies: A practical guide. Radiographics.

[3] Spanemberg J.C., Cardoso J.A., Slob E.M.G.B., López-López J. (2019). Quality of life related to oral health and its impact in adults. J. Stomatol. Oral Maxillofac. Surg..

[4] Moca A.E., Țig I.A., Ciavoi G., Iurcov R., Șipoș L.R., Todor L. (2022). The impact of the COVID-19 pandemic on the dental emergency service from Oradea, Romania: A retrospective study. Healthcare.

[5] Qin X., Zi H., Zeng X. (2022). Changes in the global burden of untreated dental caries from 1990 to 2019: A systematic analysis for the Global Burden of Disease study. Heliyon.

[6] Sicca C., Bobbio E., Quartuccio N., Nicolò G., Cistaro A. (2016). Prevention of dental caries: A review of effective treatments. J. Clin. Exp. Dent..

[7] Agnihotry A., Thompson W., Fedorowicz Z., van Zuuren E.J., Sprakel J. (2019). Antibiotic use for irreversible pulpitis. Cochrane Database Syst. Rev..

[8] Naved N., Umer F., Khowaja A.R. (2024). Irreversible pulpitis in mature permanent teeth: A cost-effectiveness analysis of pulpotomy versus root canal treatment. BMC Oral Health.

[9] Cope A.L., Francis N., Wood F., Thompson W., Chestnutt I.G. (2024). Systemic antibiotics for symptomatic apical periodontitis and acute apical abscess in adults. Cochrane Database Syst. Rev..

[10] Ryan P., McMahon G. (2012). Severe dental infections in the emergency department. Eur. J. Emerg. Med..

[11] Majewski M., Kostrzewska P., Ziółkowska S., Kijek N., Malinowski K. (2022). Traumatic dental injuries—Practical management guide. Pol. Merkur. Lekarski.

[12] Reddy L.V., Bhattacharjee R., Misch E., Sokoya M., Ducic Y. (2019). Dental injuries and management. Facial Plast. Surg..

[13] National Executive Office of the Romanian College of Dentists Recommendations for Preventing the Spread of COVID-19 in Emergency Dental Activity. https://cmdr.ro/download-zone-preview/0/1585254764/3114/7c1a06790c87eb490b59e77515bababd.

[14] Giudice A., Antonelli A., Bennardo F. (2020). To test or not to test? An opportunity to restart dentistry sustainably in the ‘COVID-19 era’. Int. Endod. J..

[15] WHO Director-General’s Opening Remarks at the Media Briefing. https://www.who.int/news-room/speeches/item/who-director-general-s-opening-remarks-at-the-media-briefing---5-may-2023.

[16] Comunicat de Presă din Data de 8 Martie 2022. https://www.mai.gov.ro/comunicat-de-presa-313/.

[17] World Health Organization Statement on the Fifteenth Meeting of the International Health Regulations (2005) Emergency Committee Regarding the Coronavirus Disease (COVID-19) Pandemic. https://www.who.int/news/item/05-05-2023-statement-on-the-fifteenth-meeting-of-the-international-health-regulations-(2005)-emergency-committee-regarding-the-coronavirus-disease-(covid-19)-pandemic.

[18] World Health Organization Oral Health Profile of Romania. https://cdn.who.int/media/docs/default-source/country-profiles/oral-health/oral-health-rou-2022-country-profile.pdf.

[19] Edlibi W.A.H., Dascălu C.G., Balcoș C., Agop-Forna D., Forna N.C. (2022). Trends in access to oral health care among adults from the N-E region of Romania. Medicina.

[20] Opydo-Szymaczek J., Borysewicz-Lewicka M., Andrysiak K., Witkowska Z., Hoffmann-Przybylska A., Przybylski P., Walicka E., Gerreth K. (2021). Clinical consequences of dental caries, parents’ perception of child’s oral health and attitudes towards dental visits in a population of 7-year-old children. Int. J. Environ. Res. Public Health.

[21] Hell C.L., Deschner J., Cores Ziskoven P., Mildenberger P., Weusmann J. (2022). Interplay of pandemic and seasonal parameters in dental emergency service. BMC Oral Health.

[22] Huang C.L., Yeh I.J., Lin Y.C., Chiu C.F., Du J.K. (2022). Analysis of adult dental emergencies at a medical center in southern Taiwan. J. Dent. Sci..

[23] Tibúrcio-Machado C.S., Michelon C., Zanatta F.B., Gomes M.S., Marin J.A., Bier C.A. (2021). The global prevalence of apical periodontitis: A systematic review and meta-analysis. Int. Endod. J..

[24] Donnermeyer D., Dammaschke T., Lipski M., Schäfer E. (2023). Effectiveness of diagnosing pulpitis: A systematic review. Int. Endod. J..

[25] Pérez A.S., Bolado E.C., Camacho-Aparicio L.A., Hervert L.P. (2023). Prevalence of pulp and periapical diseases in the endodontic postgraduate program at the national autonomous University of Mexico 2014–2019. J. Clin. Exp. Dent..

[26] Lorduy M.C., Marrugo S.P., Hernández K., Gómez L.A. (2018). Epidemiology and prevalence of pulp and periapical pathologies. Salud Uninorte.

[27] Wu K., Li C., Yang Z., Yang S., Yang W., Hua C. (2021). Changes in the characteristics of dental emergencies under the influence of SARS-CoV-2 pandemic: A retrospective study. BMC Oral Health.

[28] Zhang L., Waselewski M., Nawrocki J., Williams I., Fontana M., Chang T. (2023). Perspectives on dental health and oral hygiene practice from US adolescents and young adults during the COVID-19 pandemic. PLoS ONE.

[29] Tudoroniu C., Popa M., Iacob S.M., Pop A.L., Năsui B.A. (2020). Correlation of caries prevalence, oral health behavior, and sweets nutritional habits among 10 to 19-year-old Cluj-Napoca Romanian adolescents. Int. J. Environ. Res. Public Health.

[30] Soh N.H.B.C., Jeevanandan G., Balasubramaniam A. (2021). Prevalence of irreversible pulpitis among male and female children—A retrospective study. J. Contemp. Issues Bus. Gov..

[31] Jonson-Reid M., Wideman E. (2017). Trauma and very young children. Child Adolesc. Psychiatr. Clin. N. Am..

[32] Theriault H., Bridge G. (2023). Oral health equity for rural communities: Where are we now and where can we go from here?. Br. Dent. J..

[33] Castillo K.B., Echeto L., Schentrup D. (2023). Barriers to dental care in a rural community. J. Dent. Educ..

[34] Bencze Z., Mahrouseh N., Andrade C.A.S., Kovács N., Varga O. (2021). The burden of early childhood caries in children under 5 years old in the European Union and associated risk factors: An ecological study. Nutrients.

[35] Meyer F., Enax J. (2018). Early childhood caries: Epidemiology, aetiology, and prevention. Int. J. Dent..

[36] Altıntaş E. (2022). Complications of dental infections due to diagnostic delay during COVID-19 pandemic. BMJ Case Rep..

[37] Bennardo F., Antonelli A., Barone S., Figliuzzi M.M., Fortunato L., Giudice A. (2020). Change of Outpatient Oral Surgery during the COVID-19 Pandemic: Experience of an Italian Center. Int. J. Dent..

